# Skin Biopsy as a Diagnostic Tool for Synucleinopathies

**DOI:** 10.7759/cureus.47179

**Published:** 2023-10-17

**Authors:** Sara Waqar, Hajra Khan, Syeda K Zulfiqar, Adeel Ahmad

**Affiliations:** 1 Pathology, Geisinger Health System, Danville, USA; 2 Medicine, Rawalpindi Medical University, Rawalpindi, PAK; 3 Internal Medicine, Mayo Hospital, Lahore, PAK; 4 Dermatopathology/Dermatology/Pathology, Private Practice, Beckley, USA

**Keywords:** dementia with lewy bodies, multiple system atrophy, parkinson' s disease, skin biopsy, alpha-synucleinopathies, alpha-synuclein

## Abstract

Studies published in the last decade identified skin biopsies as a promising source of material for detecting alpha-synuclein (αSN). Alpha-synuclein gets deposited in the skin of patients with synucleinopathies, and therefore, a skin biopsy can be used to diagnose and confirm these diseases histopathologically. A skin biopsy can also be helpful for studies focusing on the nature of αSN deposits. The most important aspects of a biomarker are sensitivity, specificity, and technical feasibility. The potential for a skin biopsy to become the clinical tool of choice as a reliable biomarker for diagnosing synucleinopathies appears to be high, with consistently high sensitivity (>80%) and specificity approaching 100%. The review aims to provide an overview of the factors impacting skin biopsy's sensitivity, specificity, and feasibility in detecting dermal αSN deposits.

## Introduction and background

Skin biopsies are recommended for various dermatological diseases where histopathologic knowledge is required to diagnose the underlying skin disease [1]. Besides skin disorders, interestingly, it can also be used to identify neurological conditions called "synucleinopathies." Synucleinopathies include Parkinson's disease (PD), multiple system atrophy (MSA), dementia with Lewy bodies (DLB), pure autonomic failure (PAF), and rapid eye movement (REM) sleep behavior disorder (RBD) [2].

More than two million people in the United States are affected by synucleinopathies [3]. Depending on the location of the lesions, synucleinopathies are characterized by a chronic and progressive decrease in motor, cognitive, behavioral, and autonomic abilities [2]. These disorders and atypical parkinsonism, such as progressive supranuclear palsy (PSP) and corticobasal syndrome (CBS), share the cardinal parkinsonian symptoms [4]. This clinical overlap makes differential diagnosis sometimes exceedingly challenging [2]. Consensus clinical criteria are currently used to diagnose Parkinson's disease [3]. Even among specialists, early diagnosis of synucleinopathies has only moderate accuracy, especially in atypical and complex presentations [3]. The pathogenesis of the disease starts many years before its clinical manifestations. Motor symptoms in PD are reported to first develop when >50% of substantia nigra dopamine neurons are destroyed. Therefore, there is a much higher need for an earlier or prodromal-stage diagnostic tool that is more precise [5].

In synucleinopathies, fibrillary deposits of the alpha-synuclein (αSN) protein start to deposit in the submucosal neurons of the colon in the early phases of synucleinopathies. Besides the colon, the skin, genitourinary tract, salivary glands, and heart are also involved [5]. The skin is the most accessible organ among the peripheral tissues that have been investigated and can be used for both single and repetitive sampling. Furthermore, a skin biopsy is risk-free and minimally invasive. As a result, as various researchers have suggested, detecting and quantifying αSN deposition in skin biopsy samples may be useful for synucleinopathies [5]. This article will explore the recent publications on cutaneous αSN deposition in synucleinopathies to define its significance as a prospective biomarker for these disorders.

## Review

Methods

We have done a thorough literature search in PubMed, Google Scholar, and ResearchGate. The medical subject headings (MeSH) terms and strategy used for PubMed search were (("alpha-synuclein"[MeSh] AND "synucleinopathies"[MeSh]) AND ("biopsy"[MeSh] OR "pathology"[MeSh] )) AND "skin"[MeSh]. All articles published in PubMed in English since 2008 are included in the search criteria. For other databases, keywords used were synucleinopathies, Parkinson's disease, REM sleep behavioral disorder, multiple system atrophy, dementia with Lewy bodies, pure autonomic failure, and skin biopsy.

Discussion

After describing the importance of α-synuclein in the pathogenesis of synucleinopathies, we will discuss the role of skin biopsy in diagnosing these disorders.

Role of α-Synuclein

One of the important molecules in developing synucleinopathies is αSN. It is a classic protein found in soluble cytosolic fractions of the brain and primarily at presynaptic terminals [5]. Recent research suggests that it cooperates with the cysteine-string protein, which has a characteristic domain for heat shock protein (HSP) 40-type molecular cochaperones, to play an important role in synaptic functions [6]. In its natural (or native) state, αSN affects presynaptic signaling, membrane trafficking, and neuronal membrane integrity [7]. Lewy bodies, Lewy neurites, and glial cytoplasmic inclusions are pathological markers for the misfolding of αSN. This misfolding causes it to polymerize into fibrils and accumulate throughout the nervous system due to environmental factors like neurotoxins, low pH, high temperature, and genetic mutations [5].

Alpha-synuclein within Lewy bodies goes through different posttranslational modifications, for example, phosphorylation, cross-linking, or ubiquitination. These modifications may cause the αSN to aggregate and contribute to disease pathogenesis in vivo [8,9]. When phosphorylated at the serine 129 residue phosphorylated alpha-synuclein (p-αSN)), it leads to αSN aggregation, which has increased toxicity and leads to the development of PD [9].

Alpha-Synuclein in Cutaneous Autonomic Nerves 

**Figure 1:**
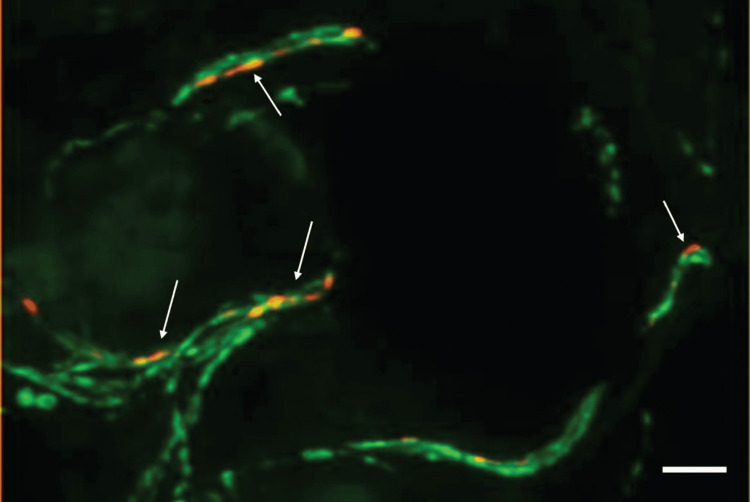
Photomicrograph of a skin biopsy of Parkinson’s disease The skin biopsy sections were cut 20-µm thick, fixed with 4% paraformaldehyde before cryopreserving, and stained with double immunofluorescence stains (anti-protein-gene-product 9.5, green, Zytomed Systems, 1:1000, and anti-p-αSN, red, Covance, 1:500). Intraaxonal p-αSN deposits are indicated with the arrows. Reprinted from Journal of Parkinson's Disease, vol. 11, Kathrin Doppler, Detection of Dermal Alpha-Synuclein Deposits as a Biomarker for Parkinson's Disease, pp. 937-947, Copyright 2021, with permission from IOS Press. The publication is available at IOS Press at http://dx.doi.org/10.3233/JPD-202489 [13] p-αSN: phosphorylated alpha-synuclein

Gibbons et al. (2009) and Wang et al. (2011) reported novel methods for studying cutaneous autonomic innervation in skin biopsies from patients with peripheral nerve disease [10,11]. Based on the prominent autonomic manifestations of Parkinson's disease, Wang et al. (2013) hypothesized that αSN deposition would be elevated in cutaneous structures with autonomic innervation [12]. They concluded that in PD patients, αSN deposition increases in cutaneous sympathetic adrenergic and sympathetic cholinergic fibers but not in the sensory fibers. Higher αSN deposition is linked to more autonomic dysfunction and advanced PD. These findings suggest that measuring αSN deposition in cutaneous autonomic nerves could be a useful biomarker in Parkinson's disease patients [12]. Figure 1 shows a photomicrograph of a double immunostained skin biopsy [13].

Studies Evaluating Skin Biopsies

Ikemura et al. obtained the first impressive outcomes on using p-αSN detection in skin biopsies as a biomarker. This study investigated the samples from autopsies and showed 70% sensitivity and 100% specificity for patients with PD compared to patients without central nervous system (CNS) Lewy body pathology [14]. Miki et al. found a very low sensitivity (10%) in biopsy samples of living patients with PD [15]. In the last decade, more studies have determined 100% specificity and 55% to 100% sensitivity compared to controls [16, 17]. Table 1 summarizes the studies done since 2010 using living subjects’ skin biopsies to detect dermal p-αSN [4,12,15-43].

**Table 1:** Summary of the studies using skin biopsies of living subjects as a diagnostic marker for synucleinopathies These studies can be compared based on their sample size, site of biopsies, type of embedding, staining techniques, sensitivity, and specificity [4,12,15-44]. PD: Parkinson’s disease; HC: healthy control; n-αSN: native alpha-synuclein; p-αSN: phosphorylated alpha-synuclein; ser 129: serine 129; double-immunofluorescence is double-labeling with an axonal marker (mostly protein gene product 9.5-PGP9.5); MSA: multiple system atrophy; PAF: pure autonomic failure; PFA: paraformaldehyde; iRBD: isolated REM sleep behavior disorder; PAN: peripheral autonomic neuropathy; DßH: dopamine-ß-hydroxylase; VIP: vasoactive intestinal polypeptide; DLB: dementia with Lewy bodies; OH: orthostatic hypotension; GBA: glucocerebrosidase gene; TH: tyrosine hydroxylase; E46K SNCA: E46K mutation in α-synuclein gene; RT-QuIC: real-time quaking-induced conversion; PSP: progressive supranuclear palsy; CBS: corticobasal syndrome; ET: essential tremor; PMCA: protein misfolding cyclic amplification

Study/ Authors	Number of Subjects	Diameter and Biopsy Sites	Embedding	Staining Used	Sensitivity (%)	Specificity (%)
Giannoccaro et al., 2022 [4]	PSP = 18; CBS = 8; PD= 26; Controls=26	3 mm, two samples each from the cervical C7 paravertebral area, thigh, and distal leg	Cryo	Double immunostained overnight with a panel of primary antibodies, including rabbit monoclonal p-SN at Ser 129 or mouse p-SN, mouse, or rabbit PGP.	53.8% for the leg site, 61.5% for the thigh, and 84.5% for the cervical area	N/A
Wang et al., 2013 [12]	20 PD, 14 HC	3 mm, distal leg, proximal, and distal thigh	Cryo	Double immunofluorescence, native αSN	N/A	N/A
Miki et al., 2010 [15]	20 PD	6 mm, chest wall, and lower limb	Parrafin	Immunostaining, p-αSN Ser 129	10	N/A
Donadio et al., 2016 [16]	16 PD, 14 PAF	3 mm, C8, thigh, and distal leg (two biopsies taken from each site)	Cryo	Double immunofluorescence, p-αSN Ser129, n-αSN	p-αSN: 100 PD and PAF, n-αSN: 100	n-αSN: 0; p-αSN: 100
Liu et al., 2020 [17]	90 PD, 30 HC	C7, thigh, distal leg, forearm, 3 mm	Cryo	Double immunofluorescence, p-αSN Ser129	83.3	100
Donadio et al., 2013 [18]	12 acquired PAN, nine PAF, and 15 HC	3 mm, distal leg and thigh, and cervical paravertebral area C8	Cryo	Double-immunostained with a panel of primary antibodies, including mouse p-αSN at Ser 129, rabbit DßH, VIP, or PGP	All PAF patients stained positive for p-αSN. No deposits in HC or acquired PAN	N/A
Doppler et al., 2015 [19]	30 PD, 12 MSA, 15 tauopathies, 39 HC	5 mm, distal, and proximal leg, Th12	Cryo	Double immunofluorescence, p-αSN Ser129	75 MSA, 73 PD	100
Haga et al., 2015 [20]	38 PD, 13 MSA	6 mm, chest wall, and lower limb	Cryo	Double immunofluorescence, p-αSN Ser129	5.3 PD, 0 MSA	N/A
Navarro-Otano et al., 2015 [21]	six PD, six HC	3 mm, distal leg	Parrafin	Immunostaining, n- and p-αSN Ser129	0	0
Zange et al., 2015 [22]	10 PD, 10 MSA, six ET	3 mm, forearm	Parrafin	Immunostaining, p-αSN Ser129	0 MSA, 100 PD	100
Antelmi et al., 2017 [23]	12 iRBD, 55 HC	3 mm, C7, and leg (2x)	Cryo	Double immunofluorescence, p-αSN Ser129	iRBD: 75	100
Donadio et al., 2017 [24]	18 DLB, 23 other dementia, 25 HC	3 mm, C8, thigh, and distal leg (two biopsies on each site)	Cryo	Double immunofluorescence, p-αSN Ser129	100	p-αSN: 100, n-αSN: 0
Donadio et al., 2017 [25]	28 PD	3 mm, C7 (2x) or C7, and Th12	Cryo	Double immunofluorescence, p-αSN Ser129	100	100
Doppler et al., 2017 [26]	18 iRBD, 25 PD, 20 HC	5 mm, distal, and proximal leg, Th12, C7	Cryo	Double immunofluorescence, p-αSN Ser129	iRBD: 55.6, PD: 80	100
Donadio et al., 2018 [27]	15 PD, 12 DLB, five PAF, 12 MSA, and 10 HC	3 mm, C7, and distal and proximal leg (two from each site)	Cryo	Double immunofluorescence, native αSN, p-αSN Ser129, and other posttranslational modifications	p-αSN: 75 for MSA, 100 for PD, PAF, and DLB	100
Donadio et al., 2018 [28]	14 subjects with neurogenic OH (PD+OH) and 14 patients with no OH (PD-OH)	3 mm, C7, and distal and proximal leg (two from each site)	Cryo	Double immunostained rabbit monoclonal p-αSN at Ser 129 or mouse p-αSN, mouse or rabbit PGP, rabbit TH, and rabbit VIP	p-αSN deposits were markedly higher in PD+OH (90% of all analyzed skin samples) than PD-OH (38%; corrected p	N/A
Doppler et al., 2018 [29]	10 PD-GBA	5 mm, distal and proximal leg, Th10, C7	Cryo	Double immunofluorescence, p-αSN	60	100
Melli et al., 2018 [30]	19 PD, 13 other parkinsonism, and 17 HC	3 mm, C8, thigh, and distal leg (two biopsies from each site)	Cryo	Double immunofluorescence, p-αSN, and aggregated αSN 5G4	p-αSN: 56, 5G4:81	p-αSN: 100, 5G4:96
Antelmi et al., 2019 [31]	30 iRBD, 17 RBD with narcolepsy	3 mm, C7, leg (two from each site)	Cryo	Double immunofluorescence, p-αSN	iRBD: 86.7	100 (compared to narcolepsy)
Kuzkina et al., 2019 [32]	27 PD, nine MSA, and 21 HC	5 mm, distal and proximal leg, Th10, C7	Cryo	Double immunofluorescence, p-αSN, truncated αSN, aggregated αSN (5G4)	p-αSN: 82; others lower (but stored sections)	100
Al-Qassabi et al., 2020 [33]	28 iRBD, 20 PD, 10 atypical parkinsonism, 21 HC	3 mm, C8	Paraffin	Double immunofluorescence, p-αSN Ser129	82 iRBD, 70 PD, 20 atypical parkinsonism	100
Chahine et al., 2020 [34]	58 PD, 21 HC	3 mm, cervical, mid-thigh (2x)	Paraffin	Immunostaining, n-αSN with Proteinase K	24	100
Donadio et al., 2020 [35]	25 PD + OD, and 25 MSA	3 mm, C7, thigh, and leg (two biopsies from each site)	Cryo	Double-immunofluorescence, p-αSN Ser129	PD + OD: 100 MSA: 72	N/A
Donadio et al., 2014 [36]	20 PD, 20 other parkinsonism, 20 HC	3 mm, C8, thigh, distal leg (two biopsies from each site)	Cryo	Double immunofluorescence, p-αSN Ser129	100	100
Doppler et al., 2014 [37]	31 PD, 35 HC	5 mm, distal, proximal leg, Th12, finger	Cryo	Double immunofluorescence, p-αSN Ser129	51.6	100
Carmona-Abellan et al., 2020 [38]	7 E46K-SNCA carrier (three DLB, two PAF, one PD, one asymptomatic) two PARK2, two HC	4 mm, C7	Paraffin	Immunostaining, p-αSN Ser129	E46K-SNCA: 100, PARK2:50	0
Giannoccaro et al., 2020 [39]	7 DLB, 21 PD, 13 PAF, 13 MSA	C7, thigh, distal leg	Cryo	Double immunofluorescence, p-αSN Ser129	100 (DLB, PAF), 95.2 (PD), 69.2 (MSA)	N/A
Wang et al., 2020 [40]	20 PD, 21 HC (+autopsy samples)	3–5 mm, leg or cervical	N/A	RT-QuIC, PMCA analysis	PMCA: 80, RT-QuIC: 95	PMCA: 90, RT-QuIC: 100
Brumberg et al., 2021 [41]	21 PD, 21 MSA	5-mm, distal and proximal leg, back (Th10), and neck (C7)	Cryo	Double immunofluorescence labeling with mouse anti-p-αSN and rabbit anti-PGP9.5, rabbit anti-tyrosine hydroxylase, or rabbit anti-VIP, and appropriate secondary antibodies	To separate PD from MSA by using either p-alpha-syn deposits in autonomic structures (28.6%) or negativity for p-alpha-syn in somatosensory fibers (90.5%)	To separate PD from MSA by using either p-alpha-syn deposits in autonomic structures (81.0%) or negativity for p-alpha-syn in somatosensory fibers (47.6%)
Donadio et al., 2021 [42]	17 with PD, five with DLB, eight with probable MSA, and three with PAF; and 38 patients with non-synucleinopathies	3 mm, proximal (C7 paravertebral) and distal sites (thigh and leg)	Cryo	Double-immunostained with a panel of primary antibodies, including rabbit monoclonal p-αSN at Ser 129 and mouse pan-neuronal marker protein gene product 9.5	90	100
Mammana et al., 2021 [43]	In vitam: 15 DLB, 13 PD, 41 controls; postmortem: one DLB, one PD, seven idiopathic LBD, 40 LBD.	In the postmortem group, thigh and cervical 3‐mm punches, and in the pre-mortem group from a single site: thigh 22 patients, cervical 23 patients. A second 3‐mm punch from the cervical region in 17 patients, the leg in 21 patients, and the thigh in 18 patients.	N/A	Skin α‐SN RT-QuIC	89.2	96.3
Wang et al., 2020 [44]	29 PD, 21 HC	3 mm, distal leg, and proximal and distal thigh	Cryo	Double immunofluorescence, free-floating for 50μm, conventional for 10/20μm, p-αSN Ser129	10μm: 73, 50μm: 100, 20μm: 90	100

Effect of Fixation and Staining Techniques on the Sensitivity of the Test

Table 1 shows that some studies detected dermal αSN in skin biopsies and demonstrated very low sensitivities for this diagnostic tool [20, 21]. Discussing potential causes, including using various biopsy protocols, fixation, immunostaining, and neuropathological evaluation, revealed the need for methodological studies comparing various protocols [17,45]. Only a few studies compare fixation and staining techniques [17,45] despite the successful demonstration of the inter- and intra-laboratory reproducibility of skin section analysis [46]. An improved protocol for paraffin sections was developed recently in the Systemic Synuclein Sampling Study (S4). This study used paraffin-embedded samples and reported skin biopsies to be 24.1% sensitive. This sensitivity is significantly lower than determined in previous studies. The highest sensitivity, however, was primarily reported in studies using cryosections when examining published studies more closely (Table 1). Unfortunately, formalin-fixed paraffin-embedded tissue was the only type of biopsy procedure systematically compared in the S4 study, even though it appears to be more practical in clinical practice [47]; this could lead to lower sensitivity (Table 1).

However, a recent study showed 70% sensitivity in PD, proving that moderate to high sensitivity is achievable when using paraffin sections [33]. This study used double-staining with an axonal marker and protease anine phosphatase pretreatment. It also analyzed more sections in cases with a fewer number of positive fibers, all of which could potentially lead to increased sensitivity [33].

Effect of Sample Thickness on Sensitivity of the Test

In various studies, the detection rate of cutaneous p-αSN in patients with PD ranged from 30% to 100% [44]. Therefore, Wang et al. (2020) hypothesized that these variations happen due to variations in the thickness of the tissue sections used for testing [44]. So, in this study, the samples were cut into 10-, 20-, and 50-µm-thick sections. 50 µm double-immunostained skin biopsy tissue sections outperformed 20 and 10 µm in detecting p-αSN in PD patients. This result can be attributed to increased tissue volume for analysis and better visualization of nerve fiber architecture [44]. 

*Selecting the Ideal Biopsy Location* 

The best biopsy site selection is another factor that might directly impact sensitivity. The leg and the C7 and C8 paravertebral regions were the sites selected in most studies (Table 1). However, only a few extensive studies systematically compare various biopsy sites. In PD, proximal locations may be involved more than distal ones [27,36,37]. However, MSA affects the distal locations more than the proximal ones [27]. The distribution of p-αSN in MSA may differ from that in PD, but larger studies are required [13].

Specificity of Dermal P-αSN as a Diagnostic Marker 

Studies that used p-αSN-specific antibodies reported a specificity of 100% compared to controls, indisputably [34,36,37]. Discrete p-αSN was only found in patient samples. Diffuse or granular p-αSN staining was reported in controls, possibly because no alkaline phosphatase pretreatment was done. [33]. 

Wang et al. (2013) reported that skin biopsies from patients with PD had higher immunoreactivity when using an antibody against native αSN than controls. However, native αSN was also found in dermal nerve fibers from healthy subjects [12]. In other studies, native αSN was similarly found in the dermal annexes' innervation of patients with synucleinopathies and controls [16,27]. Contrarily, it has also been reported that using protein K digestion or antibodies directed specifically against aggregated αSN (5G4) can distinguish between biopsies from patients with PD and controls [30,32,47].

Dermal p-αSN deposition, on the other hand, is not a specific marker of idiopathic PD and has been observed in other synucleinopathies. Phosphorylated alpha-synuclein was found primarily in somatosensory nerve fibers in MSA, as opposed to autonomic fibers in idiopathic PD [27]. Phosphorylated alpha-synuclein is frequently found in dermal autonomic nerve fibers in patients with PAF and DLB [16,24].

Feasibility of Skin Biopsy as a Diagnostic Tool

The usability of skin biopsy in diagnosing idiopathic PD is frequently debated. It is less expensive and widely available than iodine-123-radiolabeled 2β-carbomethoxy-3β-4-iodophenyl-N-3-fluoropropyl nortropane with single-photon emission computed tomography (FP-CIT-SPECT). A skin biopsy is easier than endoscopic gastrointestinal and salivary gland biopsies; patients generally tolerate it well [48]. However, laboratory equipment is required for biopsies' processing and cryoconservation. Serial sections and analyses of multiple biopsy sites are needed due to the low number of p-αSN deposits, which is time-consuming. Evaluating skin sections under the microscope requires experienced examiners and takes time, too [47].

As a result, we need high-throughput analyzing techniques that can easily be repeated on many samples in a short time. The most promising approach is real-time quaking-induced conversion (RT-QuIC), an aggregation assay created to detect prions in Creutzfeldt-Jakob disease [49]. Because alpha-synuclein contains prion-like seeding activity, small amounts of dermal αSN can be detected using RT-QuIC [49]. Several studies [49-51] found αSN aggregates in patients with idiopathic PD, dementia with Lewy bodies, and idiopathic RBD in the cerebrospinal fluid. This detection method has shown promising results on skin tissues [40,52]. We need more extensive studies with a larger sample size to fully analyze the feasibility of dermal RT-QuIC in assessing idiopathic PD.

A recent study compared immunofluorescence with RT-QuIC and found that both have high diagnostic accuracy. This study concluded that immunofluorescence has optimal reproducibility compared to RT-QuIC [42].

Limitations

This review provides an update on the studies done over the last decade to detect p-αSN in skin biopsies for diagnosing synucleinopathies. These studies are done on limited subjects; therefore, more studies with large sample sizes and standardized methodological protocols are needed to provide an accurate answer to the sensitivity, specificity, accuracy, and precision of skin biopsy as a diagnostic marker for synucleinopathies. 

## Conclusions

The discovery of p-αSN in skin biopsies from patients with synucleinopathies occurred more than 10 years ago. Several studies have confirmed that autonomic nerve fibers are involved in αSN pathology, and they can be detected in the nerve fibers with high sensitivity and specificity even in the early stages of the disease. Most of these studies were performed on Parkinson's disease patients. Current challenges are that serial skin biopsy sections must be used and stained with an immunofluorescence staining technique. Multiple skin sites must be biopsied to gain higher sensitivity on the test. All the studies have used different fixation, embedding, and sectioning techniques. Larger studies comparing these different techniques can help develop a more standardized protocol for skin biopsy to be used as a reliable biomarker for detecting synucleinopathies.
